# Supplementary material for: Omnidirectional and Broadband Antireflection Effect with Tapered Silicon Nanostructures Fabricated with Low-Cost and Large-Area Capable Nanosphere Lithography
Source: Micromachines (Basel). 2021 Jan 23;12(2):119. doi: 10.3390/mi12020119 (PMC7911543; doi:10.3390/mi12020119)
Supplement: Supplementary file 1 [file micromachines-12-00119-s001.pdf]

Supplementary File

Omnidirectional and broadband antireflection effect with tapered silicon nanostructures fabricated with low-cost and large-area-capable nanosphere lithography process

Sangho Kim, Gwan Seung Jeong, Na Yeon Park and Jea-Young Choi

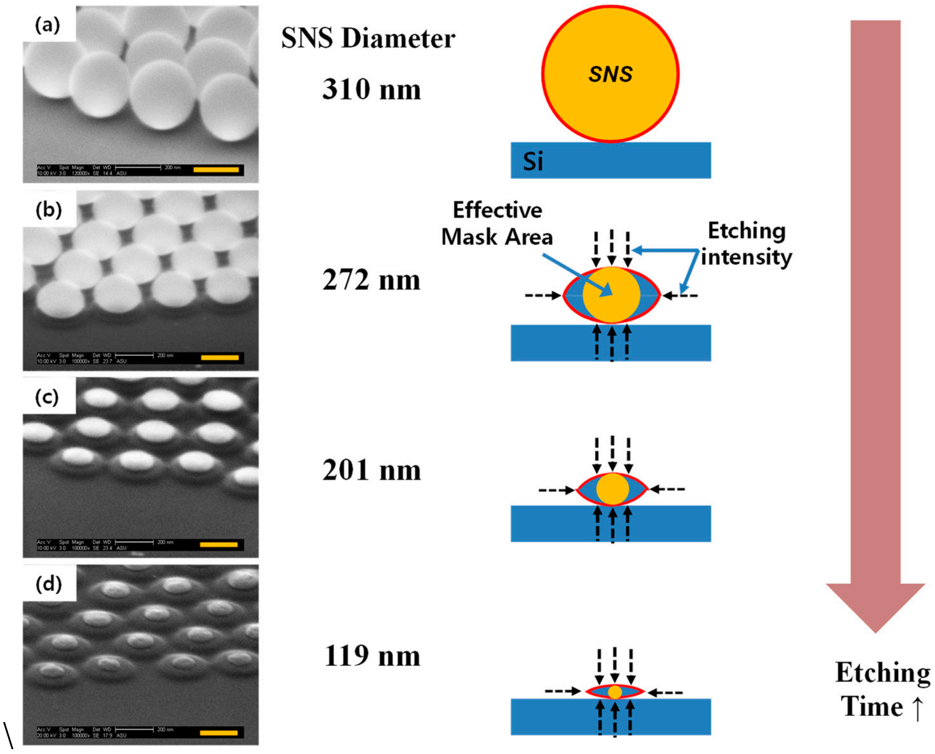

Figure S1. SNS mask size and shape change with increasing CHF_3/Ar etching time.

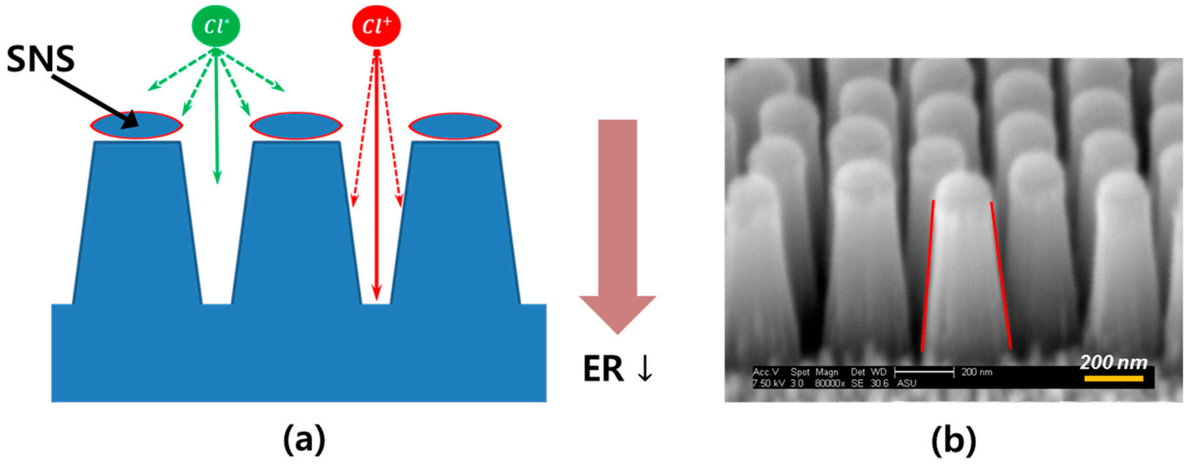

Figure S2. (**a**) Schematic illustration on aspect-ratio-dependent etching (ARDE) originated from fewer etching species reaching the bottom of high-aspect-ratio Si NPs followed by reduced ER and (b) 5-min Cl_2 etched sample with 201 nm SNS mask size.

© 2021 by the authors. Submitted for possible open access publication under the terms and conditions of the Creative Commons Attribution (CC BY) license (http://creativecommons.org/licenses/by/4.0/).
